# On the Neuroprotective Role of Astaxanthin: New Perspectives?

**DOI:** 10.3390/md16080247

**Published:** 2018-07-24

**Authors:** Christian Galasso, Ida Orefice, Paola Pellone, Paola Cirino, Roberta Miele, Adrianna Ianora, Christophe Brunet, Clementina Sansone

**Affiliations:** 1 Department of Marine Biotechnology, Stazione Zoologica Anton Dohrn, Villa Comunale, 80121 Naples, Italy; christian.galasso@szn.it (C.G.); ida.orefice@szn.it (I.O.); paola.pellone@gmail.com (P.P.); robertamiele90@libero.it (R.M.); adrianna.ianora@szn.it (A.I.); 2 Department of Research Infrastructures for Marine Biological Resources, Stazione Zoologica Anton Dohrn, Villa Comunale, 80121 Naples, Italy; paola.cirino@szn.it

**Keywords:** astaxanthin, neuroinflammation, neuroprotective effect, neuroactive carotenoids, neurodegenerative diseases

## Abstract

Astaxanthin is a carotenoid with powerful antioxidant and anti-inflammatory activity produced by several freshwater and marine microorganisms, including bacteria, yeast, fungi, and microalgae. Due to its deep red-orange color it confers a reddish hue to the flesh of salmon, shrimps, lobsters, and crayfish that feed on astaxanthin-producing organisms, which helps protect their immune system and increase their fertility. From the nutritional point of view, astaxanthin is considered one of the strongest antioxidants in nature, due to its high scavenging potential of free radicals in the human body. Recently, astaxanthin is also receiving attention for its effect on the prevention or co-treatment of neurological pathologies, including Alzheimer and Parkinson diseases. In this review, we focus on the neuroprotective properties of astaxanthin and explore the underlying mechanisms to counteract neurological diseases, mainly based on its capability to cross the blood-brain barrier and its oxidative, anti-inflammatory, and anti-apoptotic properties.

## 1. Introduction

Carotenoids have gained scientific and commercial interest during the last decades, due to their huge chemical diversity (about 750 carotenoids have been characterized) and their strong beneficial effects on human health and wellbeing. These bioactive compounds exert antioxidant, repairing, antiproliferative, antiaging and anti-inflammatory effects and can be used either as skin photo-protection to inhibit adverse effects of solar UV radiation or as nutraceutical and cosmeceutical ingredients to prevent oxidative stress-related diseases and chronic inflammation [1,2,3].

**Figure 1:**
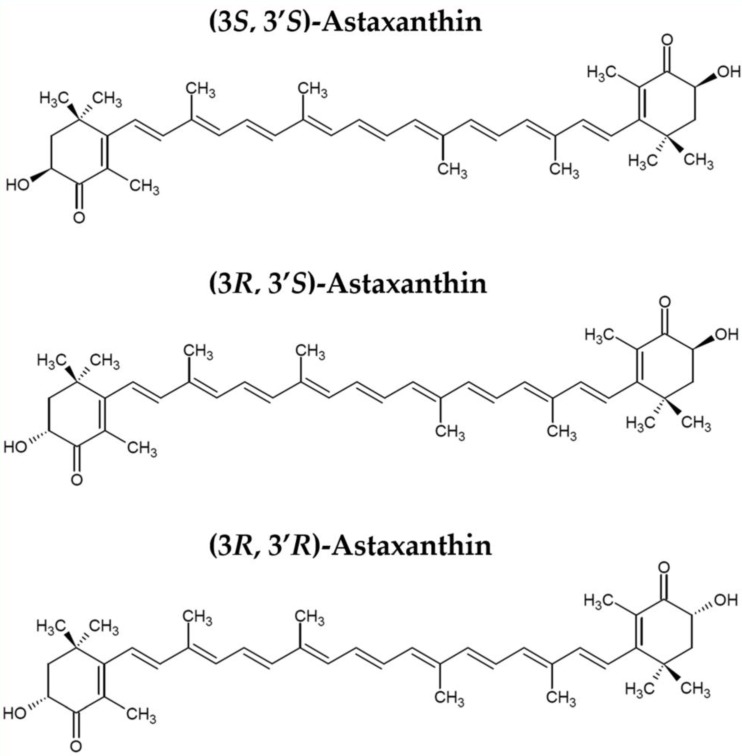
Stereoisomers of astaxanthin.

Astaxanthin is one of the most successful carotenoids on the market (Figure 1), since many studies in recent years have demonstrated its inhibitory role against oxidative stress and inflammation, dangerous processes at the basis of many chronic diseases. Moreover, astaxanthin exerts a strong protective effect on human brain; its unique chemical structure allows it to readily cross the blood-brain barrier (BBB) [4]. Thus, the brain is considered the most important target organ of astaxanthin.

Astaxanthin is present especially in the marine environment and is produced mainly by microorganisms such as bacteria, microalgae, and yeast; it can also be found in marine invertebrates and vertebrates [5,6,7]. The most important producers of astaxanthin are the marine bacterium *Agrobacterium aurantiacum*, the green microalgae *Haematococcus pluvialis* and *Chlorella zofingiensis*, and the red yeast *Xanthophyllomyces dendrorhous* (called also *Phaffia rhodozyma*). Animals cannot synthesize astaxanthin but can obtain it through the diet [8]. Astaxanthin is thus present in salmon, trout, shrimp, lobster, and fish eggs which confers a reddish-orange hue to these organisms. Nevertheless, the majority of astaxanthin-based products on the market are derived from its synthetic production, since its natural production is still not well standardized for industrial scale. Unfortunately, synthetic astaxanthin is significantly more inferior than algal-based astaxanthin in terms of anti-inflammatory and antioxidant properties [9].

Given that oxidative damage and increased neuro-inflammation are critically related with the pathogenesis of late-onset massive neuronal loss in neurodegenerative diseases, the neuroprotective effect of natural compounds, such as astaxanthin, has been of specific interest as co-treatments and prevention for these diseases [10]. The most common neurodegenerative diseases include Alzheimer Disease (AD), Parkinson Disease (PD), Huntington’s disease (HD), and amyotrophic lateral sclerosis (ALS) [11,12]. Although different neurodegenerative diseases can have several causative factors, they have some common characteristics such as an increase in ROS levels in neuronal cells caused by mitochondrial insults and the release of redox metals interacting with oxygen [13], resulting in neuronal cell death [14]. This can lead to an increase in protein aggregates that inflame and activate microglia cells [15]. Once neuroinflammation is chronically activated, cytokines and chemokines are released, producing an increment of oxidative stress, with dangerous detrimental effects on neurons [16].

The aim of this review is to report the most recent scientific findings on the protective and curative role of astaxanthin on human brain against neuroinflammation, oxidative stress and, more in general, on the beneficial effects for patients with neurodegenerative disorders (Table 1).

## 2. Brain Processes Involved in Neurodegeneration and Protective Effects of Carotenoids

The human Central Nervous System (CNS) contains 100 billion neuronal cells and an equal number of glia cells, such as microglia, astrocytes, and oligodendrocytes [36]. The CNS includes all nerves in the brain and spinal cord and is isolated from the other compartments of the human body through the blood-brain barrier (BBB). This barrier is fundamental to control and restrict the penetration of molecules (e.g., neurotoxins) and cells (e.g., immune cells or infectious agents) from peripheral parts of the body into the CNS. Tight junctions between cells forming the vascular endothelium at the CNS level confer to BBB its selective property.

CNS is able to activate the innate immune system in response to several forms of injuries, including trauma, infections, stroke and neurotoxins. A variety of cell types belonging to CNS, such as astrocytes, microglia, vascular cells, neutrophils, and macrophages, are involved in neuroinflammation [37]. Neuroinflammation is a local response of the CNS during several processes, such as neurodegeneration, trauma, and autoimmune disorders, that leads to innate immune cells mobilization and activation. Glia cells release cytokines, reactive oxygen species (ROS) and reactive nitrogen species (RNS), which could be harmful for neurons and oligodendrocytes when neuroinflammation is not a transient event. There is growing evidence suggesting that a long-standing chronic neuroinflammatory response can lead to neuronal damage, producing neurodegeneration via sustained accumulation of neurotoxic pro-inflammatory mediators [10]. The release of pro-inflammatory mediators, together with pro-oxidant agents, results in morphological and functional changes of intracellular organelles and contributes to the insurgence and progression of neurodegenerative pathologies. An example are mitochondria, intracellular targets of oxidative injuries, where chronic inflammation could produce mitochondrial dysfunction [38,39,40].

The CNS is considered highly vulnerable to oxidative stress and inflammation due to its low cell renewal potential and high cellular metabolism, since this organ requires about 25% of total body energy. This energy is fundamental for neuronal connection, axonal transport, and myelination, while mitochondrial activity produces a high amount of ROS. ROS are involved in several signal transduction pathways, such as survival, growth, proliferation and defense mechanisms against microbial infection. An unbalance between ROS production and endogenous mechanisms for detoxification of reactive oxygen intermediates leads to dysregulation of the above-mentioned mechanisms and consequent neurotoxicity and neurodegeneration [41,42].

In normal conditions and young brains, mitochondrial dysfunction is regulated and deleted by autophagy, a clearance mechanism able to remove intracellular components. In particular, mitophagy is a specific autophagic process, which is activated when damaged or superfluous mitochondria need to be removed. This signal leads to formation of autophagosomes with consequent degradation of unnecessary mitochondria. The efficiency of these clearance mechanisms diminishes with increasing age, with the accumulation of toxic molecules and damaged organelles [43,44,45]. Aging is considered the major risk factor for the insurgence and development of many neurodegenerative diseases, as also confirmed by studies describing the autophagy reduction in aging CNS cells [46,47,48]. For example, mitochondrial complex I (NADH dehydrogenase) dysfunction is strictly correlated to the idiopathic Parkinson’s disease phenotype; similarly, injury of complex II (succinate dehydrogenase) yields the Huntington’s disease phenotype. mtDNA mutations either inherited or caused by oxidative damage have been shown to contribute to Alzheimer’s disease pathology [37]. Loss of cognitive function is a common symptom in neurodegenerative diseases and there are few commercially available drugs that are able to reduce the occurrence of neuroinflammation.

Research on molecules with anti-neuroinflammatory effects and with protective properties against oxidative stress in neuronal cell models shows interesting results about some carotenoids [49,50]. Recently, it was demonstrated that astaxanthin attenuates cognitive disorders in *in vivo* and *in vitro* models for neurodegenerative diseases [4,51,52,53]. The first study in measuring carotenoids within brain compartments was carried out by Craft et al. [54]. This study quantified carotenoid content in five elderly brains and found a seeming preference for xanthophylls in the human brain.

In the last decade, some natural carotenoids, in particular those belonging to the xanthophyll family, such as lutein, crucin, crocetin, have been shown to have anti-neuroinflammatory and antioxidant effects [50,55]. The marine derived xanthophylls, such as fucoxanthin and astaxanthin, have anti-inflammatory effects and antioxidant activity on different cell lines [56,57]. Furthermore, astaxanthin has also been found to reduce hippocampal and retinal inflammation in streptozotocin-induced diabetic rats, alleviating cognitive deficits, retinal oxidative stress, and depression [17,58,59], while fucoxanthin exerts anti-inflammatory effects against various stimuli through Akt, NF-κB, and mitogen-activated protein kinase pathways [60].

The most common mechanism of action for marine xanthophylls is the suppression of inflammation pathways through the radical scavenging activity against oxygen-reactive species [5,61]. In particular, astaxanthin exerts protective effects in liver cells after induction of an inflammatory injury [62,63] and protects neuronal cells from oxidative stress [18,64], through the activation of specific pathways, such as HO-1/NOX2 axis [19] and Sp1/NR1 signaling [20]. Recent studies have demonstrated the beneficial effects of carotenoids for the treatment of neurodegenerative diseases, while a number of epidemiological studies have linked the consumption of a carotenoid rich diet with a decreased risk of neurodegenerative diseases in humans [65,66,67,68].

Extensive studies suggest that carotenoids may inhibit neurodegenerative diseases through a variety of molecular mechanisms [68,69]. For example, fucoxanthin treatment reduced Aβ-induced damage in a cultured cell model through several mechanisms including downregulation of apoptotic factors, inhibition of inflammatory cytokine-mediating action, and simultaneous reduction of ROS [70]. High levels of carotenoids within the brain, such as lutein and zeaxanthin, can enhance cognitive function in elderly people, exerting neuroprotection with a reduction of neuronal mortality [71]. In addition, high carotenoid concentrations in other body compartments provide protection against neurological pathologies. In particular, Dias and collaborators [72] found lower concentrations of carotenoids (lutein, lycopene and zeaxanthin) in dementia patients with respect to control subjects. Many studies confirm that astaxanthin delays or ameliorates the cognitive impairment associated with normal aging or alleviates the pathophysiology of various neurodegenerative diseases [73,74].

It is known that astaxanthin can cross the blood-brain barrier, a crucial feature for the treatment of neurodegenerative diseases with antioxidant compounds [75]. A recent study demonstrated that dietary astaxanthin accumulated in the hippocampus and cerebral cortex of rat brains after single and repeated ingestion. The accumulation of dietary astaxanthin in the cerebral cortex may affect maintenance and improvement of cognitive functions [76].

Astaxanthin pre-treatment promotes nerve cell regeneration, increasing gene expression of glial fibrillary acidic protein (GFAP), microtubule associated protein 2 (MAP-2), brain derived neurotrophic factor (BDNF) and growth-associated protein 43 (GAP-43) [21]. These proteins are involved in brain recovery. For example, GFAP is important in the repairing process after CNS injury, being involved in cell communication and functioning of the BBB [77]. MAP-2 is responsible of microtubule growth and neuronal regeneration; BDNF is involved in neuronal survival, growth, and differentiation of new neurons [78], while up-regulation of GAP-43 3 activates a protein kinase pathway, promoting neurite formation, regeneration, and plasticity [79].

## 3. Astaxanthin against Cognitive Disorders

Cognitive disorders are a group of mental health diseases that cause several effects on mental abilities, such as learning, problem solving, memory and perception. The most important cognitive disorders are delirium, dementia, and amnesia. Delirium is an acute confusional state characterized by inactivation, disorganized thinking, and confusion of space and time. Dementia is a progressive deterioration of the brain, memory impairment, confusion, and loss of concentration. Amnesia is a memory disorder characterized by loss of short-term memory that interferes with daily life.

Among cognitive disorders, chemobrain, a cognitive impairment caused by chemotherapeutic agents, is receiving increasing attention. These chemical agents could produce in cancer patients a strong reduction in the quality life, since they induce memory impairment, slow processing speed, and inability to concentrate. These cognitive dysfunctions seem to be linked to a reduction of neuronal integrity at the hippocampus and frontal system levels [80]. Recently, El-Agamy and collaborators [22] investigated the potential effect of astaxanthin as a protective compound able to drastically contrast the decline of cognitive functions induced by doxorubicin (DOX). Astaxanthin showed neuroprotection and memory-enhancing effects and was able to switch off inflammation and oxidative stress, mitigating the increase of acetylcholinesterase activity and suppressing several pro-apoptotic stimuli [22].

Astaxanthin also produces beneficial effects in other body compartments and cells, with direct repercussion on brain health. In particular, the positive influence of a diet rich in polar carotenoids, such as astaxanthin, on an abnormal accumulation of phospholipid hydroperoxides (PLOOH) in the erythrocytes of patients affected by dementia [23] has been described. PLOOH are the primary oxidation products of phospholipids and their accumulation in erythrocytes induce a reduction in oxygen transport to the brain, facilitating the progression of dementia [81,82,83]. Nakagawa and collaborators [23] described lower PLOOH levels in the erythrocytes and blood cells of patients treated with astaxanthin with respect to the control (placebo group), demonstrating that this bioactive molecule is responsible for the improvement of erythrocyte antioxidant status, which may contribute to the prevention of dementia.

Some marine food products are recommended by many medical authorities worldwide [84]. In particular, fish oil usually contains tocopherols, saturated fats, monounsaturated fats (mostly palmitoleic and oleic acids), and polyunsaturated fatty acids (PUFA, such as eicosapentaenoic acid, EPA, and docosahexaenoic acid, DHA). A balanced proportion between EPA and DHA (3:2, regularly found in natural fish oil) seems to be a key factor for beneficial effects, such as the slowing down of cognitive decline and reduced depression, whereas many other PUFAs (for instance the pro-inflammatory arachidonic acid) could be responsible for massive ROS production with consequent activation of immune cells [85]. The combination of astaxanthin and fish oil enhances the positive effect on brain health by reducing harmful effects due to PUFAs [86]. In particular, a Wistar rat fed with 1 mg/kg of astaxanthin and 1 mg/kg of fish oil presented lipid protection status at the anterior forebrain level. Moreover, Trolox equivalent antioxidant capacity (TAEC) and Ferric Reducing Antioxidant Power (FRAP) assessed in brain homogenates was found to increase in rats fed with a mix of astaxanthin and fish oil, with respect to only fish oil. A similar investigation was performed by Nolan et al. [87], who studied the beneficial effects of a mixture of xanthophyll carotenoids and fish oil (containing EPA and DHA) on the human brain. This particular functional food, probably due to its strong antioxidant potential, was able to reduce the progression of neurodegeneration, acting on memory, sight, and mood. The reduction of ROS production linked to marine functional foods, such as fish oil, represents a valid mechanism to prevent cognitive dysfunctions.

## 4. Astaxanthin against Alzheimer Disease (AD)

Alzheimer’s disease (AD) is one of the most severe chronic neurodegenerative disorders, characterized by memory impairment and cognitive dysfunction, due to neuronal loss mainly in the neocortex and hippocampus. The incidence of AD has increased dramatically in the last decades. From few cases at the beginning of the 20th century, in 2017 AD was reported to affect one out of five persons aged 65 and over worldwide [88]. This incidence increases drastically up to 40% over the age of 85. AD is the most common form of dementia, first described in 1906 by Alois Alzheimer. Nowadays there are many unknown aspects regarding the physiopathology of AD. Various theories have tried to explain the molecular mechanisms responsible for the initiation and progression of AD. Not one of these hypotheses alone is able to fully explain this complex neurodegenerative disease. Abnormal formation and aggregation in the brain of amyloid beta plaques are considered initial causes of AD [89]. Many studies have already demonstrated that the formation of these plaques is caused by an imbalance between synthesis and clearance of amyloid beta [90]. Accumulation of amyloid beta induces oxidative stress and inflammation at the neurofibrillary tangle level, inducing neuronal death stimuli in the brain of patients with AD [91,92]. Moreover, some studies have described the presence of high numbers of damaged mitochondria in the neurons of AD patients, probably due to mutations in mitochondrial DNA [93]. Oxidative stress at the expense of mitochondria occurs during the early stages of AD, suggesting a predominant role of oxidative stress for the progression of this disease [94]. For this reason, natural compounds with antioxidant and anti-inflammatory properties are recommended for preventing or reducing the progression of this specific neurodegeneration. In particular, astaxanthin is able to act against oxidative injuries, through various mechanisms, by quenching of singlet oxygen, scavenging of radicals, inhibiting lipid peroxidation, and regulating gene expression related to oxidative stress [5,95].

Astaxanthin exhibited strong anti-inflammatory effect, suppressing the expression of inflammatory mediators, such as TNF-α, PGE_2_ and IL-1β and blocking the production of nitric oxide (NO) and the NF-κB-dependent signaling pathway [24,25,96].

Similar anti-inflammatory effects of astaxanthin were described in other studies, using different experimental models. In particular, astaxanthin (50 µM) drastically reduced the release of inflammatory mediators in activated microglial cells (BV-2 cell line), through the modulation of factors involved in the NF-κB cascade (e.g., p-IKKα, p-IκBα, and p-NF-κB p65, IL-6 and MAPK) [97].

These findings validate the astaxanthin administration as an adjuvant therapy for AD, since this carotenoid is able to attenuate microglial activation and the consequent release of pro-inflammatory cytokines. This effect has positive repercussions on neuronal integrity, especially in elderly people, which tend to show increased inflammation in the brain [98].

Endogenous antioxidant enzymes lower their activity and efficiency with age. Results from recent studies support the beneficial effect of astaxanthin on activation of antioxidant mechanisms, increasing the levels or stimulating the activity of endogenous enzymes, such as superoxide dismutase (SOD) and catalase [26,99]. A recent study [27] investigated in mice the effect of astaxanthin on antioxidant enzymes expressed in major brain structures. Catalase and SOD were found highly expressed, together with a reduction in the level of glutathione, when mice were supplemented with 2 mg/Kg of astaxanthin for 1 month. Al-Amin and collaborators [27] also showed a reduction of malondialdehyde (MDA) and advanced protein oxidation product (APOP) levels in some brain areas, such as the frontal cortex, hippocampus, cerebellum and striatum, which showed a decrease in lipid peroxidation levels.

PC12 are neuronal cells from rats used as an artificial nervous system tissue model to study neurodegeneration and, in particular, AD. These cells were protected from neurotoxicity induced by 30 µM of beta-amyloid peptide, when treated with 0.1 µM of astaxanthin. This protective effect was due to caspase 3, Bax, IL-1β and TNFα, NF-κB inactivation, and suppression of ROS production [28]. Other studies have demonstrated the protective effect of astaxanthin on amyloid beta-induced generation of ROS and calcium dysregulation in primary hippocampal neurons [100] and the significant decrease of oxidative stress levels and cell death in PC12 cells injured with n-methyl-4-phenylpyridinium iodide (MPP+) [19].

Recent studies [4,101] have reported on the direct relationship between the healthy effects of astaxanthin on human brain and promotion of neurogenesis and plasticity, two processes that significantly decrease with age [29] and lead to cognitive decline among elderly people. Although the molecular process has not been completely elucidated, astaxanthin promotes neurogenesis and improves behavioral performance in hippocampal-dependent tasks; this could represent the predominant mechanism induced by astaxanthin, acting on cognitive functions and neurodegeneration caused by AD [4]. Neural stem cells exhibit higher proliferation rates and colony-forming capacity in a time- and dose dependent manner when treated with astaxanthin [30]. This result was corroborated by the upregulation of cyclin-dependent kinase 2 (CDK2), genes involved in proliferation control. Together with these pro-proliferative effects, astaxanthin showed protective properties in neural progenitor cells (NPC), significantly reducing apoptotic machinery in NPCs exposed to pro-oxidant agents [102].

## 5. Astaxanthin against Parkinson Disease (PD)

Parkinson’s disease (PD) affects 0.1–0.2% of the global population and this percentage drastically increases with age, reaching 1% incidence in persons aged 60 years and over [103]. Considering the prevalence of neurodegenerative diseases, PD is the second most common disorder. PD has been better characterized in recent years as a multisystem neurodegenerative disorder [104], with motor and non-motor features. In particular, the loss of dopaminergic neurons leads to motor symptoms, such as bradykinesia, rest tremor and rigidity, while degeneration of non-dopaminergic pathways is mainly responsible for the alteration of posture, balance and gait. Oxidative stress and inflammation at the CNS level contributes to insurgence and progression of PD. Unfortunately, there are few treatments that are able to reduce symptoms and to prevent or restore the loss of neurons in the CNS. As for other neurodegenerative diseases, carotenoids, in particular astaxanthin, can represent a valid co-adjuvant treatment for the prevention and/or delay of disease progression. Indeed, astaxanthin reduced neurotoxicity in PD mice [31], when animals were fed with astaxanthin derived from *Haematococcus pluvialis*, for four weeks. Grimmig and collaborators [4] demonstrated that astaxanthin possesses anti-inflammatory effects, attenuating microglia activation in substantia nigra and striatum. SH-SY5Y are human neuroblastoma cell line used to assess the potential antioxidant effect of pure compounds. Astaxanthin is a powerful antioxidant, contrasting the activation of ROS-mediated apoptosis in a dose dependent manner [105] in SH-SY5Y cells. This positive effect of astaxanthin is due to suppression of apoptosis, inhibition of mitochondrial abnormalities and the creation of intracellular ROS [18,105].

## 6. Astaxanthin against Amyotrophic Lateral Sclerosis (ALS)

Amyotrophic lateral sclerosis (ALS) is a lethal motor neuron disorder characterized by a progressive loss of the upper and lower motor neurons at the spinal or bulbar levels. There are two forms of ALS, sporadic- and familiar-type. The first type does not have a genetically inherited component. The mean age of onset varies from 50 to 65 years, while only in 5% of cases the age of onset is less than 30 years. However, ALS incident is most pronounced in people 80 years or older [106]. 5–10% of ALS cases have a genetic cause. Indeed, in some geographic areas, such as parts of Japan, Guam, Kii Peninsula of Japan and South West New Guinea, the incidence of this disease is 50–100 times higher than in other parts of the world [106].

The most common cause of ALS corresponds to a mutation of the gene encoding Cu/Zn superoxide dismutase 1 (SOD1). SOD1 is a dimeric ubiquitous enzyme mainly localized in the cytosol that catalyses the dismutation of superoxide radical into hydrogen peroxide (H_2_O_2_) and molecular oxygen (O_2_); it plays a pivotal role in the cellular homeostasis of ROS [107,108,109]. Mutant SOD1 has a structural instability that causes a misfolds in the mutated enzyme, which can lead to aggregation in the motor neurons within the CNS. More than 110 mutations of the SOD1 gene have been described and many of these mutants retain their enzymatic activity, suggesting the possibility of a toxic functional gain of these forms of mutated SOD1 in ALS [32,109,110]. One of the proposed mechanisms of neuronal death in ALS is the free radical accumulation resulting from oxidative stress. This may lead to oxidative damage of lipid, proteins, and nucleic acids, causing cell death. Free radicals are normally neutralized by antioxidant enzymes and nutrient derived antioxidants, such as vitamin C, vitamin E, and astaxanthin. The effect of different antioxidants in the treatment of people with ALS has been evaluated.

Isonaka and co-authors [111] investigated the use of antioxidants in cultured rat spinal neurons treated with the SOD1 inhibitor diethyldithiocarbamate (DDC). Results demonstrated that the use of DDC induced an increase of endogenous oxidative stress, inhibiting thus neurite growth. Antioxidants, such as L-ascorbic acid, L-histidine, α-tocopherol, β-carotene and astaxanthin may rescue the motor neurons injured by SOD1 inhibition. It is important to note that astaxanthin is the most powerful carotenoid for this specific effect. In fact, low concentrations of astaxanthin (100 nM) are needed to obtain a comparable effect of other antioxidant molecules (1 mM) [111].

## 7. Astaxanthin against Cerebral Ischemia/Reperfusion (IR)

Cerebral ischemia triggers an almost immediate loss of oxygen and glucose to the cerebral tissue and ultimately causes irreversible neuronal injuries in the ischemic core within few minutes from the onset. Cerebral ischemia/reperfusion (IR) injury is the tissue damage caused when blood supply begins to the brain after a period of ischemia. The lack of oxygen for a certain period in specific brain regions, creates a pathological microenvironment, in which the following restoration of blood circulation induces activation of inflammatory process and production of oxidative damage, rather than re-establishment of normal condition and cerebral functions. IR injury can lead to progressive learning and memory impairment as well as the loss of pyramidal neurons, which result in the progression of vascular cognitive impairment.

Ischemia induces an imbalance of endogenous pro-oxidants and antioxidants, and the overproduction of toxic free radicals. Abnormal levels of malondialdehyde (MVA), an important indicator of lipid peroxidation, as well as glutathione (GSH), and superoxide dismutase (SOD), both important as free radical scavengers, have been found in the brain tissue after cerebral IR [112,113]. In addition, the destruction of the normal structure of neurons consequently enhances oxidative stress damage and leads to neuronal apoptosis [114]. In particular, it has been demonstrated that loss of hippocampal pyramidal neurons after cerebral IR contributes to learning and memory dysfunction [33,115].

Recent studies have investigated the effect of astaxanthin supplementation to prevent the risk of ischemia on brain recovery after a cerebral IR injury.

For instance, Lee et al. [34] described *in vitro* and *in vivo* antioxidant and neuroprotective effects of astaxanthin. Oxygen Glucose Deprivation (OGD) was induced in neuronal cells (SH-SY5Y cell line) and such damaged cells were recovered in the presence of astaxanthin. The latter was able to recover cells from OGD injury in a dose dependent manner. Moreover, astaxanthin was able to reduce iNOS (inducible Nitric Oxide Synthase), HO-1 (Heme oxygenase-1, HSP32), and HSP70 (heat shock protein 70) protein levels, after OGD injury [34]. The same study highlighted the neuroprotective effect of astaxanthin on global cerebral ischemia in rats.

Xue and co-authors [35] evaluated the efficacy of AST treatment after repeated cerebral IR injury in a mouse model. These researchers demonstrated that astaxanthin improved learning and decreased memory impairment and neuronal damage. Astaxanthin treatment increased the number of pyramidal neurons of the hippocampus and restored normal neuron morphology. In addition, astaxanthin induced a strong antioxidant activity and an inhibitory effect on neuronal apoptosis in IR mice. Moreover, astaxanthin treatment reduced oxidative stress, restoring GHS levels and SOD activity, and significantly suppressed the concentration of MDA. Xue and co-authors [35] also highlighted a decrease in protein expression of cytochrome C (Cyt C) and caspase-3 (cleaved Caspase-3), both involved in the occurrence and development of apoptosis.

Pan and co-authors [116] demonstrated that astaxanthin was able to protect against brain injuries induced by transient cerebral ischemia in adult rats. The protective effect was proportional to the administered dose. Results of this study demonstrated that pre-treatment with astaxanthin lowered oxidative stress induced by ischemia, increasing the gene expression levels of SOD and decreasing those of MVA, that indicated a minor lipid peroxidation. In addition, astaxanthin increased gene expression of the nuclear factor erythroid 2-related factor 2 (Nef2), Heme oxygenase-1 (HO-1) and NAD(P)H quinone oxidoreductase 1 (NQO1), activating the Nrf2- antioxidant response element (ARE) signaling pathway.

Astaxanthin pre-treatment reduced the rate of cell death, increasing the gene expression of anti-apoptotic factor B-cell lymphoma 2 (Bcl-2), and reducing the gene expression of pro-apoptotic factor Bcl2-associated X (Bax). These two proteins are active mediators of apoptosis; in particular, Bcl-2 suppresses abnormal Ca^2+^ release from the endoplasmic reticulum (ER) and prevents ER Ca^2+^ depletion [117,118,119,120], while Bax is a homologous protein of Bcl-2 that promotes apoptosis. The balance between Bcl-2 and Bax plays an important role on ER Ca^2+^ homeostasis and in determining the fate of the cells during transient cerebral ischemia [121,122]. In conclusion, use of astaxanthin exerts protective and anti-apoptotic effects against IR injuries.

## 8. Conclusions

There is a growing body of evidence regarding the benefits of several molecules in a healthy diet that can help to prevent some age-related diseases. There is large public and scientific interest about natural nutritional supplements able to reduce the risk of age-related disease insurgence and to avoid the exacerbation of degenerative processes leading to death.

In the last decade the high demand by consumers for nutraceutical products containing bioactive compounds is directing research towards the discovery and development of renewable natural sources of bioactive molecules. In this framework, the marine environment represents a new and unexploited source for the identification or production of bioactive compounds. The global market for marine biotechnological products and processes is predicted to reach 4.8 billion USD by 2020, rising to 6.4 billion USD by 2025. In Europe, marine biotechnology was identified by the EU Blue Growth Strategy (2012) as an activity of high potential for the bioeconomy [123]. In this context, 1.20 billion USD was the estimated value for the global carotenoid market in 2016, which is expected to reach 1.53 billion USD by 2021, at a compound annual growth rate (CAGR) of 3.78% from 2016 to 2021 [124]. Such an economic growth related to these compounds is a direct result of the increased number of health conscious customers and a growing trend for natural products industries.

Among carotenoids that have beneficial effects on human health astaxanthin is one of the most successful compounds on the market. Its large scale cultivation mainly from the green microalga *H. pluvialis* is still considered more expensive [125,126]. The synthetic production of astaxanthin is still predominant in the market. This synthetic version of the carotenoid is wrongly thought as “a natural equivalent”. Actually, this production process leads to a mixture of astaxanthin isomers, which conferee important molecular and biological differences with respect to the natural carotenoid. In detail, natural astaxanthin (3S, 3′S) is more than 90% esterified, while the synthetic version is all free form, or unesterified. This chemical feature of the natural astaxanthin allows interactions with fatty acids to one or both ends of the compounds, with consequent high bioavailability [9].

Researchers are trying to improve the growth rate and astaxanthin yield of *H. pluvialis* by modulating several culture conditions [127]. In addition, preliminary studies have demonstrated the possibility of the natural astaxanthin production through sea urchin *Arbacia lixula* aquaculture without sacrifice them, since the natural carotenoid is accumulated in eggs [8]. For this reason, it is important to find a new and efficient process for the natural production of astaxanthin. Although the economic advantage of the synthetic way can obtain only racemic chemical formulation with a decrease of biological activity at 50%; this aspect encourages research to lower the cost and enhance the efficiency in the production of the natural compound.

## Figures and Tables

**Table 1:** In vitro and in vivo studies of biological roles of astaxanthin.

Model	Effect	Concentration	Target	Disease	Reference
Mice	Anti-inflammatory	25 mg/Kg/day	NF-κB, TNF-α	Cognitive impairment	[17]
SH-SY5Y cells	Anti-apoptotic	1 to 20 µM	6-OHDA, Casp3, Casp9, PARP	Not specific disease	[18]
PC12 cells	Antioxidant	5, 10, 20 μM	NOX2, NFR2, HO-1 Sp1/NR1	Not specific disease	[19,20]
Rats	Cell regeneration	20 mg/Kg/day	GFAP, MAP-2, BDNF, GAP-43 SOD, GSH,	Not specific disease	[21]
Rats	Neuroprotective	25 mg/kg	Not investigated	Cognitive disorders	[22]
Human	Antioxidant	6 or 12 mg/d	PLOOH	Dementia	[23]
Rats	Anti-inflammatory	1, 10 or 100 mg/Kg	TNF-α, PGE2, IL-1β	AD	[24]
BV-2 cells	Anti-inflammatory	50 µM	p-IKKα, p-IκBα, NF-κB p65, IL-6, MAPK	AD	[25]
Mice	Antioxidant	2 mg/kg	SOD, GSH, MDA, APOP	AD	[26]
PC12 cells	Antioxidant	0.1 µM	Bax, IL-1β, TNFα, NF-κB	AD	[27]
Primary hippocampal neurons	Antioxidant	0.1 µM	NFATc4, RyR2	AD	[28]
Neural Progenitor Cells (NPCs)	Pro-proliferative	5 and 10 ng/mL	PI3K, MEK, CDK2	AD	[29,30]
Mice	Neuroprotective	3 mg/kg	TH, IBA-1	PD	[4]
SH-SY5Y cells	Antioxidant	100 nM	PARP, CYTc	PD	[31]
Motor neurons	Antioxidant	100 nM	SOD1	ALS	[32]
SH-SY5Y cells and Rats	Neuroprotective	10 to 50 µM (cells) 30 mg/kg (rats)	iNOS, HSPs	IR	[33]
Mice	Neuroprotective	20 mg/kg	GHS, SOD, Cyt C, Casp3	IR	[34]
Rats	Neuroprotective	10 mg/Kg	SOD, MVA, Nef2, HO-1, NQO1	IR	[35]

## References

[1] Nichols J.A., Katiyar S.K. (2010). Skin photoprotection by natural polyphenols: Anti-inflammatory, anti-oxidant and DNA repair mechanisms. Arch. Dermatol. Res..

[2] Berthon J.Y., Nachat-Kappes R., Bey M., Cadoret J.P., Renimel I., Filaire E. (2017). Marine algae as attractive source to skin care. Free Radic. Res..

[3] Gonzalez S., Gilaberte Y., Philips N., Juarranz A. (2011). Current trends in photoprotection—A new generation of oral photoprotectors. Open Dermatol. J..

[4] Grimmig B., Kim S.H., Nash K., Bickford P.C., Douglas Shytle R. (2017). Neuroprotective mechanisms of astaxanthin: A potential therapeutic role in preserving cognitive function in age and neurodegeneration. GeroScience.

[5] Galasso C., Corinaldesi C., Sansone C. (2017). Carotenoids from marine organisms: Biological functions and industrial applications. Antioxidants.

[6] Yuan J.P., Peng J., Yin K., Wang J.H. (2011). Potential health-promoting effects of astaxanthin: A high-value carotenoid mostly from microalgae. Mol. Nutr. Food Res..

[7] Cirino P., Brunet C., Ciaravolo M., Galasso C., Musco L., Vega Fernández T., Sansone C., Toscano A. (2017). The sea urchin *Arbacia lixula*: A novel natural source of astaxanthin. Mar. Drugs.

[8] Galasso C., Orefice I., Toscano A., Vega Fernández T., Musco L., Brunet C., Sansone C., Cirino P. (2018). Food modulation controls astaxanthin accumulation in eggs of the sea urchin *Arbacia lixula*. Mar. Drugs.

[9] Capelli B., Bagchi D., Cysewski G.R. (2013). Synthetic astaxanthin is significantly inferior to algal-based astaxanthin as an antioxidant and may not be suitable as a human nutraceutical supplement. Nutrfoods.

[10] Cho K.S., Shin M., Kim S., Lee S.B. (2018). Recent advances in studies on the therapeutic potential of dietary carotenoids in neurodegenerative diseases. Oxid. Med. Cell. Longev..

[11] Barnham K.J., Masters C.L., Bush A.I. (2004). Neurodegenerative diseases and oxidative stress. Nat. Rev. Drug Discov..

[12] Hardy J., Selkoe D.J. (2002). The amyloid hypothesis of Alzheimer’s disease: Progress and problems on the road to therapeutics. Science.

[13] Lin M.T., Beal M.F. (2006). Mitochondrial dysfunction and oxidative stress in neurodegenerative diseases. Nature.

[14] Uttara B., Singh A.V., Zamboni P., Mahajan R.T. (2009). Oxidative stress and neurodegenerative diseases: A review of upstream and downstream antioxidant therapeutic options. Curr. Neuropharmacol..

[15] Wyss-Coray T., Mucke L. (2002). Inflammation in neurodegenerative disease—A double-edged sword. Neuron.

[16] Prakash A., Kumar A. (2014). Implicating the role of lycopene in restoration of mitochondrial enzymes and BDNF levels in β-amyloid induced Alzheimer’s disease. Eur. J. Pharmacol..

[17] Zhou X., Zhang F., Hu X., Chen J., Wen X., Sun Y., Liu Y., Tang R., Zheng K., Song Y. (2015). Inhibition of inflammation by astaxanthin alleviates cognition deficits in diabetic mice. Physiol. Behav..

[18] Ikeda Y., Tsuji S., Satoh A., Ishikura M., Shirasawa T., Shimizu T. (2008). Protective effects of astaxanthin on 6-hydroxydopamine-induced apoptosis in human neuroblastoma SH-SY5Y cells. J. Neurochem..

[19] Ye Q., Huang B., Zhang X., Zhu Y., Chen X. (2012). Astaxanthin protects against MPP+-induced oxidative stress in PC12 cells via the HO-1/NOX2 axis. BMC Neurosci..

[20] Ye Q., Zhang X., Huang B., Zhu Y., Chen X. (2013). Astaxanthin suppresses MPP^+^-induced oxidative damage in PC12 cells through a Sp1/NR1 signaling pathway. Mar. Drugs.

[21] Wu W., Wang X., Xiang Q., Meng X., Peng Y., Du N., Liu Z., Sun Q., Wang C., Liu X. (2014). Astaxanthin alleviates brain aging in rats by attenuating oxidative stress and increasing BDNF levels. Food Funct..

[22] El-Agamy S.A., Abdel-Aziz A.K., Wahdan S., Esmat A., Azab S. (2018). Astaxanthin ameliorates doxorubicin-induced cognitive impairment (chemobrain) in experimental rat model: impact on oxidative, inflammatory, and apoptotic machineries. Mol. Neurobiol..

[23] Nakagawa K., Kiko T., Miyazawa T., Kimura G.C.F., Satoh A., Miyazawa T. (2011). Antioxidant effect of astaxanthin on phospholipid peroxidation in human erythrocytes. Br. J. Nutr..

[24] Ohgami K., Shiratori K., Kotake S., Nishida T., Mizuki N., Yazawa K., Ohno S. (2003). Effects of astaxanthin on lipopolysaccharide-induced inflammation in vitro and in vivo. Investig. Ophthalmol. Vis. Sci..

[25] Solomonov Y., Hadad N., Levy R. (2018). The combined anti-inflammatory effect of astaxanthin, Lyc-O-Mato and Carnosic acid *in vitro* and *in vivo* in a mouse model of peritonitis. J. Nutr. Food Sci..

[26] Haider S., Saleem S., Perveen T., Tabassum S., Batool Z., Sadir S., Liaquat L., Madiha S. (2014). Age-related learning and memory deficits in rats: role of altered brain neurotransmitters, acetylcholinesterase activity and changes in antioxidant defense system. Age.

[27] Al-Amin M.M., Akhter S., Hasan A.T., Alam T., Hasan S.N., Saifullah A., Shohel M. (2015). The antioxidant effect of astaxanthin is higher in young mice than aged: A region specific study on brain. Metab. Brain. Dis..

[28] Chang C.H., Chen C.Y., Chiou J.Y., Peng R.Y., Peng C.H. (2010). Astaxanthin secured apoptotic death of PC12 cells induced by β-amyloid peptide 25–35: Its molecular action targets. J. Med. Food.

[29] Shetty G.A., Hattiangady B., Shetty A.K. (2013). Neural stem cell- and neurogenesis-related gene expression profiles in the young and aged dentate gyrus. Age.

[30] Kim J.H., Nam S.W., Kim B.W., Choi W., Lee J.H., Kim W.J., Choi Y.H. (2010). Astaxanthin improves stem cell potency via an increase in the proliferation of neural progenitor cells. Int. J. Mol. Sci..

[31] Grimmig B., Daly L., Subbarayan M., Hudson C., Williamson R., Nash K., Bickford P.C. (2018). Astaxanthin attenuates neurotoxicity in a mouse model of Parkinson’s disease. Oncotarget.

[32] Dion P.A., Daoud H., Rouleau G.A. (2009). Genetics of motor neuron disorders: new insights into pathogenic mechanisms. Nat. Rev. Genet..

[33] Kim H.A., Miller A.A., Drummond G.R., Thrift A.G., Arumugam T.V., Phan T.G., Srikanth V.K., Sobey C.G. (2012). Vascular cognitive impairment and Alzheimer's disease: Role of cerebral hypoperfusion and oxidative stress. Naunyn-Schmiedeberg's Arch. Pharmacol..

[34] Lee D.H., Lee Y.J., Kwon K.H. (2010). Neuroprotective effects of astaxanthin in oxygen-glucose deprivation in SH-SY5Y cells and global cerebral ischemia in rat. J. Clin. Biochem. Nutr..

[35] Xue Y., Qu Z., Fu J., Zhen J., Wang W., Cai Y., Wang W. (2017). The protective effect of astaxanthin on learning and memory deficits and oxidative stress in a mouse model of repeated cerebral ischemia/reperfusion. Brain Res. Bull..

[36] Azevedo F.A., Carvalho L.R., Grinberg L.T., Farfel J.M., Ferretti R.E., Leite R.E., Jacob Filho W., Lent R., Herculano-Houzel S. (2009). Equal numbers of neuronal and nonneuronal cells make the human brain an isometrically scaled-up primate brain. J. Comp. Neurol..

[37] Masgrau R., Guaza C., Ransohoff R.M., Galea E. (2017). Should we stop saying “glia” and “neuroinflammation”?. Trends Mol. Med..

[38] Sadeghian M., Mastrolia V., Rezaei Haddad A., Mosley A., Mullali G., Schiza D., Sajic M., Hargreaves I., Heales S., Duchen M.R. (2016). Mitochondrial dysfunction is an important cause of neurological deficits in an inflammatory model of multiple sclerosis. Sci. Rep..

[39] Urrutia P.J., Mena N.P., Núñez M.T. (2014). The interplay between iron accumulation, mitochondrial dysfunction, and inflammation during the execution step of neurodegenerative disorders. Front. Pharmacol..

[40] Campbell G., Mahad D.J. (2018). Mitochondrial dysfunction and axon degeneration in progressive multiple sclerosis. FEBS Lett..

[41] Beckhauser T.F., Francis-Oliveira J., De Pasquale R. (2016). Reactive oxygen species: Physiological and physiopathological effects on synaptic plasticity. J. Exp. Neurosci..

[42] Liu Z., Zhou T., Ziegler A.C., Dimitrion P., Zuo L. (2017). Oxidative stress in neurodegenerative diseases: From molecular mechanisms to clinical applications. Oxid. Med. Cell. Longev..

[43] Mecocci P., MacGarvey U., Kaufman A.E., Koontz D., Shoffner J.M., Wallace D.C., Beal M.F. (1993). Oxidative damage to mitochondrial DNA shows marked age-dependent increases in human brain. Ann. Neurol..

[44] Montine T.J., Neely M.D., Quinn J.F., Beal M.F., Markesbery W.R., Roberts L.J., Morrow J.D. (2002). Lipid peroxidation in aging brain and Alzheimer’s disease. Free Radic. Biol. Med..

[45] Sun N., Youle R.J., Finkel T. (2016). The Mitochondrial Basis of Aging. Mol. Cell.

[46] Hubbard V.M., Valdor R., Macian F., Cuervo A.M. (2012). Selective autophagy in the maintenance of cellular homeostasis in aging organisms. Biogerontology.

[47] Reevel A., Simcox E., Turnbull D. (2014). Ageing and Parkinson’s disease: Why is advancing age the biggest risk factor?. Ageing Res. Rev..

[48] Metaxakis A., Ploumi C., Tavernarakis N. (2018). Autophagy in age-associated neurodegeneration. Cells.

[49] Nicholls D.G. (2008). Oxidative stress and energy crises in neuronal dysfunction. Ann. N. Y. Acad. Sci..

[50] McGeer P.L., McGeer E.G., Yasojima K. (2000). Alzheimer disease and neuroinflammation. J. Neural Transm. Suppl..

[51] Che H., Li Q., Zhang T., Wang D., Yang L., Xu J., Yanagita T., Xue C., Chang Y., Wang Y. (2018). The effects of astaxanthin and docosahexaenoic acid-acylated astaxanthin on Alzheimer's disease in APP/PS1 double transgenic mice. J. Agric. Food Chem..

[52] Katagiri M., Satoh A., Tsuji S., Shirasawa T. (2012). Effects of astaxanthin-rich *Haematococcus pluvialis* extract on cognitive function: A randomised, double-blind, placebo-controlled study. J. Clin. Biochem. Nutr..

[53] Wu H., Niu H., Shao A., Wu C., Dixon B.J., Zhang J., Yang S., Wang Y. (2015). Astaxanthin as a potential neuroprotective agent for neurological diseases. Mar Drugs.

[54] Craft N.E., Haitema T.B., Garnett K.M., Fitch K.A., Dorey C.K. (2004). Carotenoid, tocopherol, and retinol concentrations in elderly human brain. J. Nutr. Health Aging.

[55] Sasaki M., Ozawa Y., Kurihara T., Noda K., Imamura Y., Kobayashi S., Ishida S., Tsubota K. (2009). Neuroprotective effect of an antioxidant, lutein, during retinal inflammation. Investig. Ophthalmol. Vis. Sci..

[56] Franceschelli S., Pesce M., Ferrone A., De Lutiis M.A., Patruno A., Grilli A., Felaco M., Speranza L. (2014). Astaxanthin treatment confers protection against oxidative stress in U937 cells stimulated with lipopolysaccharide reducing O_2_^-^ production. PLoS One.

[57] Zhang X.S., Zhang X., Wu Q., Li W., Wang C.X., Xie G.B., Zhou X.M., Shi J.X., Zhou M.L. (2014). Astaxanthin offers neuroprotection and reduces neuroinflammation in experimental subarachnoid haemorrhage. J. Surg. Res..

[58] Yeh P.T., Huang H.W., Yang C.M., Yang W.S., Yang C.H. (2016). Astaxanthin inhibits expression of retinal oxidative stress and inflammatory mediators in streptozotocin induced diabetic rats. PLoS One.

[59] Zhou X.Y., Zhang F., Hu X.T., Chen J., Tang R.X., Zheng K.Y., Song Y.J. (2017). Depression can be prevented by astaxanthin through inhibition of hippocampal inflammation in diabetic mice. Brain Res..

[60] Zhao D., Kwon S.H., Chun Y.S., Gu M.Y., Yang H.O. (2017). Anti-neuroinflammatory effects of fucoxanthin via inhibition of Akt/NF-κB and MAPKs/AP-1 pathways and activation of PKA/CREB pathway in lipopolysaccharide-activated BV-2 microglial cells. Neurochem. Res..

[61] Sansone C., Galasso C., Orefice I., Nuzzo G., Luongo E., Cutignano A., Romano G., Brunet C., Fontana A., Esposito F. (2017). The green microalga *Tetraselmis suecica* reduces oxidative stress and induces repairing mechanisms in human cells. Sci. Rep..

[62] Shen M., Chen K., Lu J., Cheng P., Xu L., Dai W., Wang F., He L., Zhang Y., Chengfen W. (2014). Protective effect of astaxanthin on liver fibrosis through modulation of TGF-*β*1 expression and autophagy. Mediators Inflamm..

[63] Li J., Wang F., Xia Y., Dai W., Chen K., Li S., Liu T., Zheng Y., Wang J., Lu W. (2015). Astaxanthin pretreatment attenuates hepatic ischemia reperfusion-induced apoptosis and autophagy via the ROS/MAPK pathway in mice. Mar. Drugs.

[64] Lee D.H., Kim C.S., Lee Y.J. (2011). Astaxanthin protects against MPTP/MPP+-induced mitochondrial dysfunction and ROS production *in vivo* and *in vitro*. Food Chem. Toxicol..

[65] Dai Q., Borenstein A.R., Wu Y., Jackson J.C., Larson E.B. (2006). Fruit and vegetable juices and Alzheimer’s disease: The Kame Project. Am. J. Med..

[66] Li F.J., Shen L., Ji H.F. (2012). Dietary intakes of vitamin E, vitamin C, and β-carotene and risk of Alzheimer’s disease: A meta-analysis. J. Alzheimers Dis..

[67] Kesse-Guyot E., Andreeva V.A., Ducros V., Jeandel C., Julia C., Hercberg S., Galan P. (2014). Carotenoid-rich dietary patterns during midlife and subsequent cognitive function. Br. J. Nutr..

[68] Mohammadzadeh Honarvar N., Saedisomeolia A., Abdolahi M., Shayeganrad A., Taheri Sangsari G., Hassanzadeh Rad B., Muench G. (2017). Molecular anti-inflammatory mechanisms of retinoids and carotenoids in Alzheimer’s disease: A review of current evidence. J. Mol. Neurosci..

[69] Fiedor J., Burda K. (2014). Potential role of carotenoids as antioxidants in human health and disease. Nutrients.

[70] Xiang S., Liu F., Lin J., Chen H., Huang C., Chen L., Zhou Y., Ye L., Zhang K., Jin J. (2017). Fucoxanthin inhibits β-amyloid assembly and attenuates β-amyloid oligomer-induced cognitive impairments. J. Agric. Food Chem..

[71] Johnson E.J., Vishwanathan R., Schalch W., Poon L., Wittwer J., Johnson M.A., Hausman D., Davey A., Green R., Gearing M. (2011). Brain levels of lutein (L) and zeaxanthin (Z) are related to cognitive function in centenarians. FASEB J..

[72] Dias I.H., Polidori M.C., Li L., Weber D., Stahl W., Nelles G., Grune T., Griffiths H.R. (2014). Plasma levels of HDL and carotenoids are lower in dementia patients with vascular comorbidities. J. Alzheimers Dis..

[73] Wen X., Huang A., Hu J., Zhong Z., Liu Y., Li Z., Pan X., Liu Z. (2015). Neuroprotective effect of astaxanthin against glutamate-induced cytotoxicity in HT22 cells: Involvement of the Akt/GSK-3β pathway. Neuroscience.

[74] Xu L., Zhu J., Yin W., Ding X. (2015). Astaxanthin improves cognitive deficits from oxidative stress, nitric oxide synthase and inflammation through upregulation of PI3K/Akt in diabetes rat. Int. J. Clin. Exp. Pathol..

[75] Petri D., Lundebye A.K. (2007). Tissue distribution of astaxanthin in rats following exposure to graded levels in the feed. Comp. Biochem. Physiol. C Toxicol Pharmacol..

[76] Manabe Y., Komatsu T., Seki S., Sugawara T. (2018). Dietary astaxanthin can accumulate in the brain of rats. Biosci. Biotechnol. Biochem..

[77] Cullen D.K., Simon C.M., LaPlaca M.C. (2007). Strain rate-dependent induction of reactive astrogliosis and cell death in three-dimensional neuronal-astrocytic co-cultures. Brain Res..

[78] Ahmed S., Reynolds B.A., Weiss S. (1995). BDNF enhances the differentiation but not the survival of CNS stem cell-derived neuronal precursors. J. Neurosci..

[79] Benowitz L.I., Routtenberg A. (1997). GAP-43: An intrinsic determinant of neuronal development and plasticity. Trends Neurosci..

[80] Correa D.D., Ahles T.A. (2008). Neurocognitive changes in cancer survivors. Cancer J..

[81] Ajmani R.S., Metter E.J., Jaykumar R., Ingram D.K., Spangler E.L., Abugo O.O., Rifkind J.M. (2000). Hemodynamic changes during aging associated with cerebral blood flow and impaired cognitive function. Neurobiol. Aging.

[82] Mohanty J.G., Eckley D.M., Williamson J.D., Launer L.J., Rifkind J.M. (2008). Do red blood cell-b-amyloid interactions alter oxygen delivery in Alzheimer’s disease?. Adv. Exp. Med. Biol..

[83] Leijenaara J.F., van Maurik I.S., Kuijer J.P.A., van der Flier W.M., Scheltens P., Barkhof F., Prins N.D. (2017). Lower cerebral blood flow in subjects with Alzheimer's dementia, mild cognitive impairment, and subjective cognitive decline using two-dimensional phase-contrast magnetic resonance imaging. Alzheimers Dement..

[84] Cunnane S.C., Plourde M., Pifferi F., Bégin M., Féart C., Barberger-Gateau P. (2009). Fish, docosahexaenoic acid and Alzheimer's disease. Prog. Lipid Res..

[85] Gorjão R., Azevedo-Martins A.K., Rodrigues H.G., Abdulkader F., Arcisio-Miranda M., Procopio J., Curi R. (2009). Comparative effects of DHA and EPA on cell function. Pharmacol. Ther..

[86] Mattei R., Polotow T.G., Vardaris C.V., Guerra B.A., Leite J.R., Otton R., Barros M.P. (2011). Astaxanthin limits fish oil-related oxidative insult in the anterior forebrain of Wistar rats: Putative anxiolytic effects?. Pharmacol. Biochem. Behav..

[87] Nolan J.M., Mulcahy R., Power R., Moran R., Howard A.N. (2018). Nutritional intervention to prevent Alzheimer's Disease: potential benefits of xanthophyll carotenoids and Omega-3 Fatty acids combined. J. Alzheimers Dis..

[88] Karlawisha J., Jack C.R., Rocca W.A., Snyder H.M., Carillo M.C. (2017). Alzheimer’s disease: The next frontier—Special report 2017. Alzheimers Dement..

[89] Irvine G.B., El-Agnaf O.M., Shankar G.M., Walsh D.M. (2008). Protein aggregation in the brain: The molecular basis for Alzheimer’s and Parkinson’s diseases. Mol. Med..

[90] Wildsmith K.R., Holley M., Savage J.C., Skerrett R., Landreth G.E. (2013). Evidence for impaired amyloid β clearance in Alzheimer’s disease. Alzheimers Res. Ther..

[91] Manczak M., Anekonda T.S., Henson E., Park B.S., Quinn J., Reddy P.H. (2006). Mitochondria are a direct site of Aβ accumulation in Alzheimer’s disease neurons: Implications for free radical generation and oxidative damage in disease progression. Hum. Mol. Genet..

[92] Hanzel C.E., Pichet-Binette A., Pimentel L.S., Iulita M.F., Allard S., Ducatenzeiler A., Do Carmo S., Cuello A.C. (2014). Neuronal driven pre-plaque inflammation in a transgenic rat model of Alzheimer’s disease. Neurobiol. Aging.

[93] De la Monte S.M., Wands J.R. (2006). Molecular indices of oxidative stress and mitochondrial dysfunction occur early and often progress with severity of Alzheimer’s disease. J. Alzheimers Dis..

[94] Nunomura A., Perry G., Aliev G., Hirai K., Takeda A., Balraj E.K., Jones P.K., Ghanbari H., Wataya T., Shimohama S. (2001). Oxidative damage is the earliest event in Alzheimer disease. J. Neuropathol. Exp. Neurol..

[95] Dose J., Matsugo S., Yokokawa H., Koshida Y., Okazaki S., Seidel U., Eggersdorfer M., Rimbach G., Esatbeyoglu T. (2016). Free radical scavenging and cellular antioxidant properties of astaxanthin. Int. J. Mol. Sci..

[96] Suzuki Y., Ohgami K., Shiratori K., Jin X.H., Ilieva I., Koyama Y., Yazawa K., Yoshida K., Kase S., Ohno S. (2006). Suppressive effects of astaxanthin against rat endotoxin-induced uveitis by inhibiting the NF-κB signaling pathway. Exp. Eye Res..

[97] Kim Y.H., Koh H.K., Kim D.S. (2010). Down-regulation of IL-6 production by astaxanthin via ERK-, MSK-, and NF-κB-mediated signals in activated microglia. Int. Immunopharmacol..

[98] Satoh A., Tsuji S., Okada Y., Murakami N., Urami M., Nakagawa K., Ishikura M., Katagiri M., Koga Y., Shirasawa T. (2009). Preliminary clinical evaluation of toxicity and efficacy of a new astaxanthin-rich *Haematococcus pluvialis* extract. J. Clin. Biochem. Nutr..

[99] Puertas M.C., Martinez-Martos J.M., Cobo M.P., Carrera M.P., Mayas M.D., Ramirez-Exposito M.J. (2012). Plasma oxidative stress parameters in men and women with early stage Alzheimer type dementia. Exp. Gerontol..

[100] Lobos P., Bruna B., Cordova A., Barattini P., Galáz J.L., Adasme T., Hidalgo C., Muñoz P., Paula-Lima A. (2016). Astaxanthin protects primary hippocampal neurons against noxious effects of Aβ-oligomers. Neural Plast..

[101] Yook J.S., Okamoto M., Rakwal R., Shibato J., Lee M.C., Matsui T., Chang H., Cho J.Y., Soya H. (2016). Astaxanthin supplementation enhances adult hippocampal neurogenesis and spatial memory in mice. Mol. Nutr. Food Res..

[102] Kim J.H., Choi W., Lee J.H., Jeon S.J., Choi Y.H., Kim B.W., Chang H.I., Nam S.W. (2009). Astaxanthin inhibits H_2_O_2_-mediated apoptotic cell death in mouse neural progenitor cells via modulation of P38 and MEK signaling pathways. J. Microbiol. Biotechnol..

[103] Tysnes O.B., Storstein A. (2017). Epidemiology of Parkinson’s disease. J. Neural. Transm..

[104] Archibald N., Miller N., Rochester L. (2013). Neurorehabilitation in Parkinson disease. Handb. Clin. Neurol..

[105] Liu X., Shibata T., Hisaka S., Osawa T. (2008). Astaxanthin inhibits reactive oxygen species-mediated cellular toxicity in dopaminergic SH-SY5Y cells via mitochondria-targeted protective mechanism. Brain Res..

[106] Zarei S., Carr K., Reiley L., Diaz K., Guerra O., Altamirano P.F., Pagani W., Lodin D., Orozco G., Chinea A. (2015). A comprehensive review of amyotrophic lateral sclerosis. Surg. Neurol. Int..

[107] Fridovich I. (1995). Superoxide radical and superoxide dismutases. Annu. Rev. Biochem..

[108] Russo M., Cocco S., Secondo A., Adornetto A., Bassi A., Nunziata A., Polichetti G., De Felice B., Damiano S., Serù R. (2011). Cigarette smoke condensate causes a decrease of the gene expression of Cu-Zn superoxide dismutase, Mn superoxide dismutase, glutathione peroxidase, catalase, and free radical-induced cell injury in SH-SY5Y human neuroblastoma cells. Neurotox. Res..

[109] Damiano S., Sasso A., Accetta R., Monda M., De Luca B., Pavone L.M., Belfiore A., Santillo M., Mondola P. (2018). Effect of mutated Cu, Zn superoxide dismutase (SOD1^G93A^) on modulation of transductional pathway mediated by M1 muscarinic receptor in SK-N-BE and NSC-34 cells. Front. Physiol..

[110] Strong M.J., Kesavapany S., Pant H.C. (2005). The pathobiology of amyotrophic lateral sclerosis: A proteinopathy?. J. Neuropathol. Exp. Neurol..

[111] Isonaka R., Hiruma H., Katakura T., Kawakami T. (2011). Inhibition of superoxide dismutase selectively suppresses growth of rat spinal motor neurons: comparison with phosphorylated neurofilament-containing spinal neurons. Brain Res..

[112] Zhang R., Liu C., Liu X., Guo Y. (2016). Protective effect of *Spatholobus suberectus* onbrain tissues in cerebral ischemia. Am. J. Transl. Res..

[113] Vani J.R., Mohammadi M.T., Foroshani M.S., Jafari M. (2016). Polyhydroxylated fullerene nanoparticles attenuate brain infarction and oxidative stress in rat model of ischemic stroke. EXCLI J..

[114] Park E., Choi S.K., Kang S.W., Pak Y.K., Lee G.J., Chung J.H., Park H.K. (2015). Cerebral ischemia-induced mitochondrial changes in a global ischemic rat model by AFM. Biomed. Pharmacother..

[115] Bennett S., Grant M.M., Aldred S. (2009). Oxidative stress in vascular dementia and Alzheimer's disease: A common pathology. J. Alzheimers Dis..

[116] Pan L., Zhou Y., Li X.F., Wan Q.J., Yu L.H. (2017). Preventive treatment of astaxanthin provides neuroprotection through suppression of reactive oxygen species and activation of antioxidant defense pathway after stroke in rats. Brain Res Bull..

[117] Lam M., Distelhorst C.W. (1994). Evidence that Bcl-2 represses apoptosis by regulating endoplasmic reticulum-associated Ca^2+^ fluxes. Proc. Natl. Acad. Sci. USA.

[118] Distelhorst C.W., Lam M., McCormick T.S. (1996). Bcl-2 inhibits hydrogen peroxide-induced ER Ca^2+^ pool depletion. Oncogene.

[119] He H., Lam M., Mccormick T.S., Distelhorst C.W. (1997). Maintenance of calcium homeostasis in the endoplasmic reticulum by Bcl-2. J. Cell Biol..

[120] Wei H., Wei W., Bredesen D.E., Perry D.C. (1998). Bcl-2 protects against apoptosis in neuronal cell line caused by thapsigargin-induced depletion of intracellular calcium stores. J. Neurochem..

[121] Antonsson B., Conti F., Ciavatta A., Montessuit S., Lewis S., Martinou I., Bernasconi L., Bernard A., Mermod J.J., Mazzei G., Maundrell K. (1997). Inhibition of Bax channel-forming activity by Bcl-2. Science.

[122] Antonsson B., Montessuit S., Lauper S., Eskes R., Martinou J.C. (2000). Bax oligomerization is required for channel-forming activity in liposomes and totrigger cytochrome c release from mitochondria. Biochem. J..

[123] European Marine Board and Marine Biotechnology (ERANET) (2017). Marine Biotechnology: Advancing Innovation in Europe’s Bioeconomy. EMB Policy Brief.

[124] Markets and Markets Website—New Market Reports. http://www.marketsandmarkets.com/search.asp?Search=carotenoid&x=0&y=0.

[125] Raja R., Hemaiswarya S., Kumar N.A., Sridhar S., Rengasamy R. (2008). A perspective on the biotechnological potential of microalgae. Crit. Rev. Microbiol..

[126] Misawa N. (2009). Pathway engineering of plants toward astaxanthin production. Plant Biotechnol..

[127] Cheng J., Li K., Yang Z., Zhou J., Cen K. (2016). Enhancing the growth rate and astaxanthin yield of *Haematococcus pluvialis* by nuclear irradiation and high concentration of carbon dioxide stress. Bioresour. Technol..

